# Issues in accelerometer methodology: the role of epoch length on estimates of physical activity and relationships with health outcomes in overweight, post-menopausal women

**DOI:** 10.1186/1479-5868-7-53

**Published:** 2010-06-15

**Authors:** Kelley Pettee Gabriel, James J McClain, Kendra K Schmid, Kristi L Storti, Robin R High, Darcy A Underwood, Lewis H Kuller, Andrea M Kriska

**Affiliations:** 1 Division of Epidemiology and Disease Control; University of Texas Health Science Center; Austin, TX 78701, USA; 2 Cancer Prevention Fellowship Program; National Cancer Institute; Bethesda, MD 20852, USA; 3 Department of Biostatistics; University of Nebraska Medical Center; Omaha, NE 68198-4375, USA; 4 Department of Epidemiology; University of Pittsburgh; Pittsburgh, PA 15261, USA

## Abstract

**Background:**

Current accelerometer technology allows for data collection using brief time sampling intervals (i.e., epochs). The study aims were to examine the role of epoch length on physical activity estimates and subsequent relationships with clinically-meaningful health outcomes in post-menopausal women.

**Methods:**

Data was obtained from the Woman On the Move through Activity and Nutrition Study (n = 102). Differences in activity estimates presented as 60s and 10s epochs were evaluated using paired t-tests. Relationships with health outcomes were examined using correlational and regression analyses to evaluate differences by epoch length.

**Results:**

Inactivity, moderate- and vigorous-intensity activity (MVPA) were significantly higher and light-intensity activity was significantly lower (all *P *< 0.001) when presented as 10s epochs. The correlation between inactivity and self-reported physical activity was stronger with 10s estimates (*P *< 0.03); however, the regression slopes were not significantly different. Conversely, relationships between MVPA and body weight, BMI, whole body and trunk lean and fat mass, and femoral neck bone mineral density was stronger with 60s estimates (all *P *< 0.05); however, regression slopes were similar.

**Conclusion:**

These findings suggest that although the use of a shorter time sampling interval may suggestively reduce misclassification error of physical activity estimates, associations with health outcomes did not yield strikingly different results. Additional studies are needed to further our understanding of the ways in which epoch length contributes to the ascertainment of physical activity in research studies.

**Trial Registration:**

Clinical Trials Identifier: NCT00023543

## Background

Waist-worn accelerometers provide a reliable and valid objective measure of free-living physical activity[1]. Accelerometers have been used in many research applications including validation of self-report physical activity measures, identification of psychosocial and environmental correlates of physical activity behavior, quantification of physical activity to examine relationships with health outcomes, measurement of physical activity levels within a population-based surveillance system [i.e., National Health and Nutrition Examination Survey (NHANES)], and evaluation of the success of physical activity or lifestyle interventions [1-3]. As accelerometers become increasingly more popular to ascertain physical activity levels in research settings, further development and refinement of methodologies related to data reduction and analyses are needed.

Many accelerometers function by integrating a filtered acceleration signal over a user-defined time sampling interval, which is commonly referred to as an epoch. At the end of each epoch, the summed value (i.e., activity count) is stored in the monitor memory[4]; this process is repeated until data collection is complete. First generation accelerometers had limited data storage capabilities; therefore, research applications that required continuous data collection over an extended period of time (i.e., 7 days) were limited to the use of a 60s (second) epoch. Technological advancements have increased the memory capacity of accelerometers, which permit researchers to make use of a shorter time sampling interval. However, the benefit of utilizing a shorter time sampling interval when compared to collecting physical activity data using the more conventional 60s epoch is not fully understood[5].

To date, much of the accelerometer-based research that utilized shorter epoch intervals has been done in children. When compared with adults, children tend to engage in frequent bursts of moderate- to vigorous- intensity physical activity (MVPA) that last over a relatively short period of time[6,7]. Therefore, capturing this pattern of frequent, high-intensity, short duration physical activity is optimized with a shorter sampling interval. If the traditional 60s epoch was used in children, shorter bursts of MVPA would simply be averaged over the minute and would likely go undetected[4]. Regardless of age, the imprecise detection of true physical activity levels increases the risk for non-differential misclassification, which can result in a reduction in the strength of association between physical activity and health outcomes of interest.

Little is known about the role of epoch length on adult estimates of physical activity[4]. It is intuitive that capturing activity in shorter epochs would result in a more precise measurement of overall physical activity. However, it is unknown whether this level of precision will significantly enhance our understanding of the association between measured physical activity and health outcomes. Therefore, the objectives of the current study are to: 1) compare physical activity estimates collected over shorter epoch intervals (i.e., 10s) with physical activity estimates that were generated using traditional time sampling intervals (i.e., 60s) and 2) examine whether physical activity data presented as 10s, rather than 60s epochs, improved the ability to evaluate physical activity-health outcome relationships. These objectives were examined using cross-sectional data from the 48 month follow-up visit of the Woman On the Move through Activity and Nutrition (WOMAN) Study, a randomized clinical trial of primary cardiovascular disease (CVD) prevention in overweight, post-menopausal women. In women, the risk of chronic disease increases exponentially as they transition through menopause[8-10]; therefore, examining the role of epoch length on health outcomes that are common among this population sub-group is imperative to inform best practices for prevention. Accelerometer data was collected using 10s epoch in order to utilize data processing methods that were available during the time of the 48 month visit.

## Methods

### Study participants and design

The WOMAN Study was designed to evaluate the effectiveness of a non-pharmacological lifestyle intervention focused on weight loss through dietary and physical activity changes to reduce measures of sub-clinical atherosclerosis among early postmenopausal women aged 52-62 years. The study design of the WOMAN Study, including a description of the health education and lifestyle change groups has been previously reported[11]. Briefly, eligibility criteria for study enrollment included body mass index (BMI) between 25-39.9 kg/m^2^, average waist circumference (WC) ≥80 cm, no diagnosed CVD, type 2 diabetes mellitus, or psychotic disorder including depression, blood pressure ≤ 140/90 mmHg with or without antihypertensive therapy, low density lipoprotein cholesterol (LDL-c) level between 100-160 mg/dL without lipid lowering therapy, and completion of the 400 m walk test. Women were recruited through direct mailing from selected ZIP codes in Allegheny County, Pennsylvania from April 2002 to October 2003. The study protocol was approved by the institutional review board at the University of Pittsburgh and all participants provided written informed consent.

### Participant characteristics

Age and demographic factors including race/ethnicity, educational attainment, marital status, and health behavior information were collected using standardized questionnaires. Information on medication use (i.e., hormone, lipid lowering, antihypertensive, and hypoglycemic therapy) was obtained via self-report and from a medication inventory.

### Physical activity

Accelerometer data was collected using the ActiGraph GT1M accelerometer (Pensacola, FL). The ActiGraph GTIM accelerometer is a small (3.8 × 3.7 × 1.8cm), uni-axial piezoelectric activity monitor that measures acceleration in the vertical plane. Data output from the ActiGraph accelerometer are activity counts (ct), which quantify the amplitude and frequency of detected accelerations, and activity counts are summed over a user-specified time interval (i.e., epoch). The sum of the activity counts over an epoch is related to activity intensity and can be categorized based on validated activity count cut-points[1,3,12]. In the current study, data were collected in 10s epochs and data were reintegrated into 60s epochs for comparison. Technical specifications, as well as reliability and validity of the ActiGraph[1,13] have been described previously.

WOMAN Study participants were instructed to wear the accelerometer on a belt over the hip corresponding to their dominant hand for seven consecutive days during all waking hours. Data from the accelerometer were downloaded and screened for wear time using methods reported by Troiano et al[14]. Average activity volume (ct/min/d) was calculated using summed daily counts detected over wear periods. Time spent per day (min/d) in different intensity levels was estimated using threshold values obtained from prior calibration studies and used in the 2003-2004 NHANES analyses by Troiano et al[3]. For classification of 10s epochs, NHANES threshold values were multiplied by a factor of 0.17 (i.e., 10s/60s). Summary accelerometer-determined physical activity estimates were averaged (per day) for all participants with at least four valid days of 10 or more hours of wear time.

Self-reported physical activity was collected using the past-year version of the Modifiable Activity Questionnaire (MAQ), an interviewer-administered questionnaire, which assesses leisure and occupational activities over the past year[15]. Due to the limited reported occupational activity in the WOMAN Study population[16], only the leisure physical activity estimate is reported. Physical activity levels were calculated as the product of the duration and frequency of 39 common leisure activities (hr·wk^-1^), weighted by a standardized estimate of the metabolic equivalent (MET) of each activity[17], and then summed for all activities performed. Self-reported leisure physical activity is expressed as metabolic equivalent hours per week (MET∙hr∙wk^-1^). The MAQ has been previously shown to be a reliable[15,18] and valid[15,18,19] estimate of self-reported physical activity.

### 400 meter (w) walk

The 400 m walk test is a component of the long distance corridor walk protocol that requires participants to walk 10 laps along a hallway with cones set 20 meters apart[20]. Participants with elevated blood pressure (BP ≥ 200/110 mmHg), resting heart rate (HR) (>110 or <40 bpm), or self-reported chest pain, shortness of breath, or cardiac event or procedure within the past three months were excluded for safety reasons. The 400 m walk was stopped if the participant's HR exceeded 135 bpm or if chest pain or dyspnea was reported. Data is expressed as the time (s) taken to complete the 400 m distance. The 400 m walk has been shown to be reliable and significantly associated with measured maximal oxygen consumption in middle-aged women[21].

### Bone mineral density and body composition

BMI was calculated from height and weight measured with a stadiometer and calibrated balance beam scale by dividing the participant's weight in kilograms by the square of her height in meters. Average WC was measured at the navel (horizontal plane at the center of the navel) using a fiberglass retractable tape measure. A Hologic QDR 4500 W densiometer (Hologic, Inc.; Bedford, MA) was used to ascertain total hip, femoral neck, and whole body areal bone mineral density (BMD, g/cm^2^). Lumbar spine BMD was determined from the subregional lumbar spine BMD in the whole-body scan. Total bone mineral-free lean and total fat mass were also derived from the whole-body scan. Dual energy x-ray absorptiometry (DXA) quality assurance measurements were conducted to ensure scanner reliability.

### Cardiovascular disease risk factors

Total cholesterol, high density lipoprotein cholesterol (HDL-c), triglycerides, and glucose were determined by conventional methods. LDL-c was estimated by the Friedewald equation and insulin was measured via radioimmunoassay. Blood pressure was measured using a pulse-obliteration procedure. Briefly, prior to blood pressure measurement, the participant's arm was measured for appropriate cuff size. Participants then sat quietly in a room for 5 minutes with both feet flat on the floor and then pulse-obliteration pressure was obtained. The cuff was then inflated to a level of approximately 40 mmHg above the pulse-obliteration level and deflated at a rate of 2 mmHg per s while listening for Korotkoff sounds. After a brief period, blood pressure was taken a second time and the average of the two readings was recorded.

### Statistical methods

Univariate analyses were conducted on all relevant measured parameters. All variables were assessed for normality and examined for potential outliers. When examining the distributions of the variables, self-reported physical activity and insulin levels each had two leverage points that were deemed outliers and removed from the analyses. Normally distributed variables were reported as mean and standard deviation, non-normal data as medians with interquartile range, and proportions were noted for categorical variables. Differences in measured parameters between randomized groups were evaluated using Student t-tests, Wilcoxon Rank Sum, chi square (χ^2^) or Fisher's exact tests.

#### Study objective #1: comparison of physical activity data obtained using 60s vs. 10s epochs

Paired t-tests were used to compare the physical activity estimates by epoch interval utilized. The agreement between categories (i.e., quartiles) of physical activity estimates and meeting versus not meeting *2008 Physical Activity Guidelines*, defined as 150 min/wk of MVPA [22], expressed as 60 and 10s epochs were then evaluated using Kendall's tau -b with 95% confidence intervals.

#### Study objective #2: evaluation of 60 vs. 10s epochs within a research setting

Scatterplots between accelerometer-derived physical activity estimates and health outcomes were examined for linearity and Pearson's product-moment correlation coefficients were used to describe the linear relationship between health outcomes and accelerometer-determined data presented as 10 and 60s epochs[23]. Hypothesis tests for dependent correlations were performed to determine whether the strength of the relationships between health outcome and physical activity varied by epoch length[24,25]. Linear mixed models (i.e., repeated measures regression) were used to quantify the rate of increase or decrease in health outcome per unit increase in physical activity for 10 and 60s epochs. Tests of equality between the two slopes were then computed to determine if measuring the rate of increase or decrease in health outcome differed based on epoch length[26]. Correlation coefficients, including tests evaluating the difference in strength of correlations[25], and regression models were repeated after additional adjustment for BMI for all health outcomes except for anthropometric measures due to high colinearity. All statistical analyses were generated using SAS/STAT software, Version 9.2 of the SAS System for Windows (Cary, NC).

## Results

A total of 508 women met the eligibility criteria for the study and were randomized to either the health education or lifestyle change group using a block randomized design. Of the 508, 454 participants completed the clinic portion of the 48 month follow-up visit. At this visit, 257 women were randomly approached to take part in an accelerometer sub-study; of which 102 (39.7%) agreed to participate. When compared to women who were not part of the accelerometer sub-study, women who wore the accelerometer had significantly lower BMI [29.0 (3.9) vs. 30.5 (4.3) kg/m^2^; *P *= 0.0008] and average WC [98.1 (11.0) vs. 100.9 (11.6) cm; *P *= 0.03] and higher self-reported leisure physical activity [15.9 (9.0, 24.3) vs. 10.8 (5.0, 19.6) MET∙hr∙wk^-1^; *P *= 0.0005] at the 48 month follow-up visit.

The characteristics of the study participants are presented in Table 1. The mean age of participants included in this report at the 48 month follow-up was 61.0 (2.8) years and most were white, non-smokers, and achieved at least a high school degree. Few differences were noted in demographic factors, anthropometrics, accelerometer-derived physical activity, or health outcomes between randomized groups at the 48 month follow-up visit. However, women in the lifestyle change group had higher self-reported leisure physical activity, lower BMI and insulin, and fewer initiated lipid lowering therapy when compared to the health education group (all *P *< 0.05). It is important to note that the formal intervention ended at approximately 36 months; therefore, it is not surprising that the randomized groups were similar at 48 months. Accordingly, the following results are presented in the full sample.

**Table 1:** Participant characteristics in the Women on the Move through Activity and Nutrition (WOMAN) Study (n = 102).

	WOMAN Study Participants
**Demographic Factors**	
Age at 48 month visit, years	61.0 (2.8)
High school graduate, %	98.0
Non-White, %	5.9
Married, %	66.7
Current smoker at 48 month visit, %	1.0
Medication Use at 48 month visit, %	
Hormone therapy	16.7
Lipid lowering therapy	10.8
Hypertensive therapy	38.2
Hypoglycemic therapy	1.0
**Anthropometric Measures**	
Body weight, lbs	169.7 (25.7)
Body Mass Index, kg/m^2^	29.0 (3.9)
Waist Circumference, cm	98.1 (11.0)
Whole body fat mass, kg	30.9 (6.9)
Trunk fat mass, kg	14.6 (3.9)
Whole body lean mass, kg	45.6 (5.5)
Trunk lean mass, kg	22.4 (2.8)

**Bone Parameters**	

Spine, g/cm^2^	0.99 (0.14)
Trochanter, g/cm^2^	0.70 (0.11)
Intertrochanter, g/cm^2^	1.09 (0.14)
Femoral Neck, g/cm^2^	0.78 (0.10)
Hip, g/cm^2^	0.92 (0.12)

**Physical Activity**	

Leisure Physical Activity, MET∙hr∙wk^-1^	15.7 (9.0, 24.2)
400 m walk, s	306.7 (39.8)

**Cardiovascular Disease Risk Factors**	

Systolic Blood Pressure, mmHg	122.0 (116.0, 134.0)
Diastolic Blood Pressure, mmHg	78.0 (8.1)
Total Cholesterol, mg/dL	215.6 (33.6)
LDL-c, mg/dL	125.0 (30.7)
HDL-c, mg/dL	64.6 (53.6, 77.6)
Triglycerides, mg/dL	107.0 (82.0, 152.0)
Insulin, mg/dL	12.8 (4.7)
Glucose, mg/dL	102.1 (10.6)

Continuous data presented as mean (standard deviation) or median (interquartile range); categorical data as proportions (%).

### Study objective #1: comparison of physical activity data obtained using 60 vs. 10s epochs

WOMAN Study participants wore the ActiGraph accelerometer for 865.2 (70.9) min/d and average physical activity volume was 315.7 (114.4) ct/min/d. Table 2 presents descriptive accelerometer data expressed as 60 and 10s epochs for physical activity summary estimates that included every minute above a threshold level as well as time spent in bouts of MVPA lasting at least 8 of 10 minutes (i.e., modified 10 minute bout)[3]. The absolute difference in physical activity estimates by epoch interval is also presented. When compared to physical activity estimates derived using 60s epochs, the mean time spent per day in inactivity, moderate- and vigorous-intensity physical activity were significantly higher and light-intensity physical activity was significantly lower when the 10s sampling interval was utilized (all *P *< 0.001).

**Table 2:** Accelerometer data^† ^expressed using 60 and 10 second (s) epoch intervals in WOMAN Study Participants at the 48 month follow-up visit (n = 102).

	60s epoch		10s epoch		Δ^††^	t-value
	*Mean (SD)*	*Range*	*Mean (SD)*	*Range*		
Inactivity: wear time^†††^	.60 (0.08)	.36 - .79	.72 (0.07)	.50 - .86	-.11 (0.02)	-50.3*
Inactivity (0-99 ct), min/day	523.4 (79.0)	351.2 - 702.9	622.8 (71.2)	449.2 - 765.5	-99.4 (22.4)	-44.9*
Light Intensity (100-<2020 ct), min/day	315.0 (74.2)	167.1 - 572.9	200.1 (51.8)	98.8 - 388.8	114.9 (27.0)	43.0*
Moderate Intensity (2020-<5999 ct), min/day	26.1 (19.6)	.28 - 92.4	41.2 (21.7)	3.7 - 107.0	-15.0 (5.9)	-25.5*
Vigorous Intensity (≥5999 ct), min/day	.7 (2.8)	.05 - 15.7	1.2 (3.0)	.04 - 17.9	-.5 (0.63)	-8.1*
MVPA (≥2020 ct), min/day	26.9 (20.1)	.28 - 92.9	42.4 (22.4)	3.8 - 109.5	-15.5 (6.1)	-25.8*
Activity Bouts of MVPA (≥2020 ct), min/day	14.6 (16.3)	0 - 70.4	14.6 (16.3)	0 - 70.4	---	---

^†^Data presented as mean (standard deviation)

^††^Δ calculated as the difference between the mean time spent per day in a given intensity level using a 60 second epoch interval (i.e., current practice) minus the average time spent per day in a given intensity level using a 10 second epoch interval and then averaged for all participants.

^†††^Presented as the ratio between inactivity and wear time.

*Paired t-tests were used to test the hypothesis of no difference in physical activity summary estimates between ActiGraph data collected as 10 or 60 second epochs; *P *< 0.0001

To reframe the results presented in Table 2, agreement between quartiles of physical activity, as well as meeting vs. not meeting current physical activity recommendations, presented as 60 or 10s epochs was examined (Table 3). The Kendall's tau-b statistic was strongest with inactivity (adjusted for wear time) and weakest with moderate intensity physical activity (0.90 and 0.85, respectively). The median and 25^th ^and 75^th ^percentile values for vigorous intensity physical activity expressed as 60s epochs were zero; therefore, agreement with equivalent categories of 10s data could not be computed. Twenty-seven (26.5%) participants were classified as meeting physical activity guidelines using 10s, but not 60s, estimates of MVPA. No (0%) participants were categorized as meeting guidelines with 60s estimate of MVPA that were not also classified in this manner using 10s epoch data. The remaining participants were categorized similarly regardless of whether 10 or 60s epoch data was utilized [Kendall tau-b: 0.53 (0.42, 0.65)].

**Table 3:** Agreement between quartiles of accelerometer data^† ^expressed using 60 and 10 second (s) epoch intervals collected in WOMAN Study Participants at the 48 month follow-up visit (n = 102).

	Kendall's tau-b	95% Confidence Limits
Inactivity: wear time	0.90	0.86, 0.95
Inactivity (0-99 ct), min/day^††^	0.88	0.82, 0.93
Light Intensity (100-<2020 ct), min/day	0.87	0.82, 0.92
Moderate Intensity (2020-<5999 ct), min/day	0.85	0.79, 0.90
Vigorous Intensity (≥5999 ct) ^†^, min/day	---	---
MVPA (≥2020 ct), min/day	0.86	0.80, 0.91

Quartile ranges created using median (interquartile range) that are presented in table 1 for light-, moderate-, vigorous, and moderate- to vigorous- intensity physical activity. Quartile ranges for inactivity were <471.6, ≥471.6-<527.4, ≥527.4-<580.1, and ≥580.1 for 60 second epoch data and <583.5, ≥583.5-<632.1, ≥632.1-<665.8, and ≥665.8 for 10 second epoch data.

^†^Unable to compute, median (IQR) for vigorous- intensity physical activity collected in 60s epochs was 0 (0.0, 0.0)

^††^Presented as the ratio between inactivity and wear time.

### Study objective #2: evaluation of physical activity-health outcome relationships by epoch length

Regardless of the time sampling interval used, physical inactivity, adjusted for wear time, was inversely related to self-reported leisure physical activity and directly related to insulin levels (both *P *< 0.05), with no other significant relationships noted (Table 4). The correlation between self-reported physical activity and inactivity was significantly stronger with data collected as 10s epochs (*P *< 0.01); however, the regression slope between health outcome and inactivity expressed as 60 vs. 10s epochs were not statistically different. After additional adjustment for BMI, results were similar. However, only the correlation between physical inactivity estimate, captured via 60s epochs, and glucose and HDL-c was statistically significant (all *P *< 0.05). Similar to the unadjusted results, the correlation between past year reported physical activity and physical inactivity adjusted for BMI was significantly stronger with data collected as 10s epochs (*P *= 0.04); however, the regression slopes were not significantly different (data not shown). (NOTE: refer to Additional file 1)

**Table 4:** Relationship between inactivity [adjusted for wear time (min/day)] accumulated in 60 and 10 second epochs and health outcome measures (n = 102).

	60s epoch	10s epoch	**Test for Difference Between Correlations ***p *value	**60s epoch **β	**10s epoch **β	**Test for Equality of Slope ***p *value
	ρ	ρ				
**Anthropometric Measures**						

Body weight, lbs	-0.077	-0.077	0.98	-0.0003	-0.0002	0.56
Body Mass Index, kg/m^2^	-0.073	-0.055	0.40	-0.0015	-0.0009	0.30
Waist Circumference, cm	0.053	0.042	0.61	0.0004	0.0003	0.49
Whole body fat mass, kg	-0.011	0.014	0.24	-0.0001	0.0001	0.44
Trunk fat mass, kg	0.030	0.056	0.24	0.0000	0.0000	---
Whole body lean mass, kg	-0.037	-0.038	0.97	-0.0000	-0.0000	---
Trunk lean mass, kg	-0.008	-0.006	0.93	-0.0000	-0.0000	---

**Bone Parameters**						

Spine, g/cm^2^	-0.006	0.009	0.48	-0.0036	0.0041	0.64
Trochanter, g/cm^2^	-0.033	-0.047	0.51	-0.0244	-0.0277	0.88
Intertrochanter, g/cm^2^	0.003	0.009	0.77	0.0019	0.0044	0.88
Femoral Neck, g/cm^2^	-0.100	-0.092	0.70	-0.0781	-0.0568	0.33
Hip, g/cm^2^	-0.015	-0.013	0.92	-0.0108	-0.0074	0.86

**Physical Activity**						

Leisure Physical Activity, MET∙hr∙wk^-1 b^	-0.255*	-0.302**	0.03	-0.0014**	-0.0013***	0.51
400 m walk, s ^d^	0.031	0.048	0.43	0.0001	0.0001	0.80

**Cardiovascular Disease Risk Factors**						

Systolic Blood Pressure, mmHg	0.028	0.039	0.60	0.0002	0.0002	0.92
Diastolic Blood Pressure, mmHg	-0.016	-0.008	0.70	-0.0002	-0.0001	0.72
Total Cholesterol, mg/dL ^a^	-0.179^†^	-0.161	0.39	-0.0004^†^	-0.0003	0.07
LDL-c, mg/dL ^a^	-0.123	-0.099	0.26	-0.0003	-0.0002	0.12
HDL-c, mg/dL ^a^	-0.154	-0.148	0.76	-0.0008	-0.0006	0.18
Triglycerides, mg/dL ^a^	0.036	0.014	0.30	0.0001	0.0000	0.38
Insulin, mg/dL ^c^	0.212*	0.224*	0.57	0.0036*	0.0030*	0.23
Glucose, mg/dL ^a^	0.182^†^	0.166^†^	0.45	0.0014^†^	0.0010^†^	0.07

^†^*P *< 0.10; *P < 0.05; **P < 0.01; ***P < 0.001; ^a ^n = 101; ^b ^n = 100; ^c ^n = 99; ^d ^n = 90

Regardless of epoch length, MVPA was inversely associated with anthropometric measures including BMI, waist circumference, and whole body and trunk fat (all *P *< 0.05) (Table 5). MVPA collected every 60s was also significantly related to body weight, whole body and trunk lean mass (all *P *< 0.05), whereas the correlation between these anthropometric measures and 10s data were either borderline statistically significant or null. MVPA presented for both 60 and 10s epochs was also directly associated with spine BMD (both *P *< 0.05), self-reported past year physical activity (both *P *< 0.001) and inversely related to 400 m walk time (both *P *< 0.001), insulin (both *P *< 0.001), and glucose (both *P *< 0.05). MVPA collected in 10s epochs was also inversely related to systolic BP (*P *< 0.05); whereas, the relationship was not statistically significant when reintegrated to 60s data. The correlation between MVPA and body weight, BMI, whole body and trunk fat and lean mass were significantly stronger with data expressed using a 60s epoch when compared to the 10s data (all *P *< 0.05). However, there was no difference in correlation strength by epoch length with spine BMD, self-reported leisure physical activity, time taken to complete the 400 m walk, insulin or glucose collected at the 48 month follow-up visit. When examining the equality of the regression slopes between health outcome and MVPA by epoch interval, no significant differences were noted. Results were similar after additional adjustment for BMI; however, MVPA was no longer significantly associated with systolic BP or glucose. Finally, the association between triglycerides and MVPA was significantly stronger when expressed as 60s epochs; however, similar to the unadjusted results, there were no differences in slopes between 60 and 10s data for any health outcome (data not shown). (NOTE: refer to Additional file 2)

**Table 5:** Relationship between moderate- to vigorous- intensity physical activity (min/day) accumulated in 60 and 10 second epochs and health outcome measures (n = 102).

	60s epoch	10s epoch	**Test for Difference Between Correlations ***p *value	**60s epoch **β	**10s epoch **β	**Test for Equality of Slope ***p *value
	ρ	ρ				
**Anthropometric Measures**						

Body weight, lbs	-0.231*	-0.165^†^	0.01	-0.18*	-0.14^†^	0.12
Body Mass Index, kg/m^2^	-0.253*	-0.202*	0.05	-1.30*	-1.16*	0.36
Waist Circumference, cm	-0.286**	-0.273**	0.59	-0.52**	-0.55**	0.55
Whole body fat mass, kg	-0.334***	-0.251*	0.001	-0.96	-0.81	0.07
Trunk fat mass, kg	-0.347***	-0.286**	0.01	-1.76	-1.63	0.37
Whole body lean mass, kg	-0.198*	-0.136	0.02	-0.72*	-0.55	0.13
Trunk lean mass, kg	-0.228*	-0.177^†^	0.05	-1.64*	-1.42^†^	0.32

**Bone Parameters**						

Spine, g/cm^2^	-0.270**	-0.253*	0.51	-38.58**	-40.37*	0.68
Trochanter, g/cm^2^	-0.075	-0.045	0.27	-13.70	-9.27	0.43
Intertrochanter, g/cm^2^	-0.110	-0.083	0.31	-16.31	-13.75	0.57
Femoral Neck, g/cm^2^	-0.122	-0.063	0.02	-23.31	-13.43	0.09
Hip, g/cm^2^	-0.113	-0.080	0.20	-19.57	-15.37	0.42

**Physical Activity**						

Leisure Physical Activity, MET∙hr∙wk^-1 b^	0.336***	0.335***	0.97	0.45***	0.51***	0.18
400 m walk, s ^d^	-0.437***	-0.415***	0.40	-0.22***	-0.23***	0.45

**Cardiovascular Disease Risk Factors**						

Systolic Blood Pressure, mmHg	-0.169^†^	-0.203*	0.19	-0.22^†^	-0.29*	0.06
Diastolic Blood Pressure, mmHg	-0.101	-0.115	0.62	-0.25	-0.32	0.38
Total Cholesterol, mg/dL ^a^	0.042	0.055	0.64	0.03	0.04	0.53
LDL-c, mg/dL ^a^	0.006	0.030	0.38	0.004	0.02	0.38
HDL-c, mg/dL ^a^	0.105	0.115	0.70	0.13	0.16	0.44
Triglycerides, mg/dL ^a^	-0.040	-0.085	0.09	-0.01	-0.04	0.07
Insulin, mg/dL ^c^	-0.307**	-0.289**	0.49	-1.29**	-1.35**	0.66
Glucose, mg/dL ^a^	-0.212*	-0.213*	0.99	-0.40*	-0.45*	0.41

^†^*P *< 0.10; *P < 0.05; **P < 0.01; ***P < 0.001; ^a ^n = 101; ^b ^n = 100; ^c ^n = 99; ^d ^n = 90

## Discussion

In the current investigation, accelerometer-derived physical activity estimates and relationships with health outcomes were compared by length of time sampling interval (i.e., 60s vs. 10s epoch). Findings suggest that although there was a significant difference in physical activity estimates by epoch length, the relationship between physical activity and most health outcomes did not vary by length of data collection. There were, however, a few health outcomes that were more strongly correlated with MVPA presented as 60s epochs when compared to 10s epoch-derived estimates. This finding may relate to the underlying role of physical activity in the causation of obesity such that 60s estimates may better reflect total energy expenditure. However, when examining the equality of the regression slope in these variables by epoch length, no significant differences in relationships were noted. This suggests that measuring the rate of change in health outcomes as a function of physical activity is not dependent on the length of data collection based on current processing methods. Further, additional adjustment for BMI did not elicit strikingly different findings when compared with the unadjusted results.

The current study is not the first to observe differences of intensity classification based on epoch length, but builds upon previous research that was limited to children and adolescents[27-29]. Given the nature and primary purpose of the WOMAN Study, a criterion measure of physical activity was not included for additional comparison of study findings. Regardless, several key sources of error in physical activity outcomes based on 60s data are worthy of noting. First, intermittent activities are easily misclassified, with brief moderate intensity activities often categorized as light intensity once data are summed over a full minute. Similarly, brief periods of movement can result in misclassification of predominately sedentary minutes as light intensity. Second, sustained bouts of activity lasting at least 60s rarely start and stop in synchronization with the accelerometer's internal clock. As a result, a 60s bout of walking, or the beginning and end of a longer walking bout, can easily be split over more than one 60s interval, which could result in misclassification of moderate activities as light intensity. These sources of misclassification can result in substantial shifts between 60 and 10s epoch for minutes detected as light vs. inactive and smaller, but important, shifts between minutes detected as light vs. MVPA. Perhaps most striking is the transfer of roughly 100 min/d detected as light intensity based on 60s epochs to inactive minutes, raising the proportion of inactive or sedentary time from 60% to 72% of monitored time, or an additional 1 hour and 39 minutes of sedentary time per day among these women. Understanding the role that epoch length has in defining physical activity estimates is of critical importance in order to reduce sources of systematic error in research studies.

It is important to note that physical activity estimates were derived from activity count threshold-based processing methods that have been used almost exclusively by researchers over the past decade[1]. However, advancements in accelerometer technology have enhanced the level of sophistication of available data processing methods and interpretation of derived summary estimates. Early efforts by Crouter et al., utilized a two regression approach that predicted intensity from separate equations based on the variability of the detected activity sampled in 10s epochs[30,31]. However, as previously stated, the ActiGraph and other devices are now capable of sampling data using 1s epochs or in raw acceleration mode to provide a dense data stream for use in emerging signal processing and pattern recognition models (e.g., artificial neural networks)[2,32]. Recent progress in accelerometer processing methods offer promise for increased accuracy of physical activity estimates from body worn accelerometers; precision that could potentially improve the ability to observe significant relationships with health outcomes beyond those observed in the current study. In the current report, accelerometer data was sampled using 10s epochs in order to utilize data processing methods that were available at the time the 48 month follow-up visits were initiated. It is important to note that these results were gleaned from 2 epoch lengths and examined in a specific population sub-group. However, since high frequency accelerometer data can be reintegrated to a lower resolution, as was done in the current report (i.e., 10s to 60s epochs), with no additional burden, we would encourage researchers to collect data at the maximum allowable resolution for the desired monitoring period in order to preserve the ability to utilize the latest advancements in accelerometer data processing methods for use in epidemiological studies as they become available.

When interpreting the findings, several limitations need to be considered. Given the cross-sectional nature of the data, no inferences should be made about the causality of relationships between physical activity and health outcomes. Due to limited resources, accelerometer data was only collected in one-quarter of WOMAN Study participants who attended the 48 month follow-up visit. Although collecting accelerometer data in all participants attending the 48 month follow-up visit would have increased the power to see more significant physical activity-health outcome relationships, few differences were noted between those who were included in the accelerometer sub-study and those who were not. Finally, waist-worn, uni-axial accelerometers provide an accurate measure of predominantly ambulatory activities and, thus, do not capture all physical activities that may contribute to an improvement in health outcomes[3]. Furthermore, comparisons with self-reported leisure physical activity may also be limited as the past-year MAQ includes non-ambulatory activities, but not lower intensity activities (i.e., household chores), which may be captured with accelerometers. Therefore, the weak or null relationships between health outcome and physical activity that were observed in the current study may be a reflection of the limited quantification of total physical activity or simply the result of the relatively homogeneous nature of the study sample.

## Conclusions

In summary, findings from the current report suggest that although the use of a shorter time sampling interval may suggestively reduce misclassification error of physical activity estimates, the estimates did not elicit strikingly different associations with health outcomes. These results have important public health implications, particularly for researchers who might not have the current resources available to replace first generation accelerometers with newer models that are equipped with enhanced memory capacity. Our findings suggest that accelerometer data collected over longer epoch intervals provides meaningful physical activity estimates that relate well to a comprehensive portfolio of diverse and clinically-meaningful health outcomes and validates the findings of earlier studies that were limited to use of a 60s epoch. Since data was obtained from a select population of overweight, healthy, post-menopausal women, we would like to encourage future studies to examine these important questions in their own study populations to further our understanding of the extent to which epoch length contributes to the ascertainment of physical activity in research studies.

## Competing interests

The authors declare that they have no competing interests.

## Authors' contributions

Significant writer (KPG, JJM), significant reviewer (KKS, KLS, RRH, DAU, LHK, and AMK), manuscript concept/design (KPG, JJM, KLS, and AMK), data acquisition (KPG, DAU), data analysis (KPG, RRH), and statistical expertise (KLS, RRH). All authors have read and approved the final manuscript.

## Supplementary Material

Additional file 1 **Relationship between inactivity [adjusted for wear time (min/day)] accumulated in 60 and 10 second epochs and health outcome measures after adjustment for body mass index kg/m^2 ^(n = 102)**. Click here for file

Additional file 2 **Relationship between moderate- to vigorous- intensity physical activity (min/day) accumulated in 60 and 10 second epochs and health outcome measures after adjustment for body mass index kg/m^2 ^(n = 102)**. Click here for file
