# Supplementary material for: Issues in accelerometer methodology: the role of epoch length on estimates of physical activity and relationships with health outcomes in overweight, post-menopausal women
Source: Int J Behav Nutr Phys Act. 2010 Jun 15;7:53. doi: 10.1186/1479-5868-7-53 (PMC2900223; doi:10.1186/1479-5868-7-53)
Supplement: Additional file 1 — Relationship between inactivity [adjusted for wear time (min/day)] accumulated in 60 and 10 second epochs and health outcome measures after adjustment for body mass index kg/m2 (n = 102). [file 1479-5868-7-53-S1.DOC]

Additional file 1: Relationship between inactivity [adjusted for wear time (min/day)] accumulated in 60 and 10 second epochs and health outcome measures after adjustment for body mass index kg/m^2^ (n=102).

|  | **60s**  **epoch** | **10s**  **epoch** | **Test for Difference**  **Between Correlations**  *p* value | **60s epoch**  β | **10s epoch**  β | **Test for Equality**  **of Slope**  *p* value |
| --- | --- | --- | --- | --- | --- | --- |
|  | ρ | ρ |
| **Anthropometric Measures** |  |  |  |  |  |  |
| Body weight, lbs | --- | --- | --- | --- | --- | --- |
| Body Mass Index, kg/m^2^ | --- | --- | --- | --- | --- | --- |
| Waist Circumference, cm | --- | --- | --- | --- | --- | --- |
| Whole body fat mass, kg | --- | --- | --- | --- | --- | --- |
| Trunk fat mass, kg | --- | --- | --- | --- | --- | --- |
| Whole body lean mass, kg | --- | --- | --- | --- | --- | --- |
| Trunk lean mass, kg | --- | --- | --- | --- | --- | --- |
| **Bone Parameters** |  |  |  |  |  |  |
| Spine, g/cm^2^ | 0.012 | 0.023 | 0.60 | -0.004 | 0.004 | 0.64 |
| Trochanter, g/cm^2^ | -0.013 | -0.033 | 0.36 | -0.030 | -0.033 | 0.88 |
| Intertrochanter, g/cm^2^ | 0.028 | 0.029 | 0.99 | 0.0005 | 0.003 | 0.88 |
| Femoral Neck, g/cm^2^ | -0.077 | -0.076 | 0.97 | -0.086 | -0.065 | 0.33 |
| Hip, g/cm^2^ | 0.011 | 0.007 | 0.84 | -0.014 | -0.011 | 0.86 |
| **Physical Activity** |  |  |  |  |  |  |
| Leisure Physical Activity, MET∙hr∙wk^-1 b^ | -0.263^**^ | -0.308^**^ | 0.04 | -0.001^*^ | -0.001^**^ | 0.51 |
| 400 m walk, s ^d^ | 0.069 | 0.081 | 0.58 | 0.0001 | 0.0001 | 0.80 |
| **Cardiovascular Disease Risk Factors** |  |  |  |  |  |  |
| Systolic Blood Pressure, mmHg | 0.052 | 0.058 | 0.78 | 0.0002 | 0.0002 | 0.92 |
| Diastolic Blood Pressure, mmHg | 0.001 | 0.005 | 0.84 | -0.0002 | -0.0001 | 0.72 |
| Total Cholesterol, mg/dL ^a^ | -0.172^†^ | -0.156 | 0.42 | -0.0004^†^ | -0.0003 | 0.07 |
| LDL-c, mg/dL ^a^ | -0.111 | -0.090 | 0.31 | -0.0003 | -0.0002 | 0.12 |
| HDL-c, mg/dL ^a^ | -0.200^*^ | -0.187^†^ | 0.52 | -0.0008 | -0.0006 | 0.18 |
| Triglycerides, mg/dL ^a^ | 0.069 | 0.040 | 0.17 | 0.0001 | 0.0000 | 0.38 |
| Insulin, mg/dL ^c^ | 0.321^**^ | 0.322^**^ | 0.95 | 0.004^*^ | 0.004^**^ | 0.23 |
| Glucose, mg/dL ^a^ | 0.223^*^ | 0.200^*^ | 0.28 | 0.001^†^ | 0.001^†^ | 0.07 |

^†^*P*<0.10; ^*^*P*<0.05; ^**^*P*<0.01; ^***^*P*<0.001; ^a^ n=101; ^b^ n=100; ^c^ n=99; ^d^ n=90
