# Supplementary material for: Issues in accelerometer methodology: the role of epoch length on estimates of physical activity and relationships with health outcomes in overweight, post-menopausal women
Source: Int J Behav Nutr Phys Act. 2010 Jun 15;7:53. doi: 10.1186/1479-5868-7-53 (PMC2900223; doi:10.1186/1479-5868-7-53)
Supplement: Additional file 2 — Relationship between moderate- to vigorous- intensity physical activity (min/day) accumulated in 60 and 10 second epochs and health outcome measures after adjustment for body mass index kg/m2 (n = 102). [file 1479-5868-7-53-S2.DOC]

Additional file 2: Relationship between moderate- to vigorous- intensity physical activity (min/day) accumulated in 60 and 10 second epochs and health outcome measures after adjustment for body mass index kg/m^2^ (n=102).

|  | **60s**  **epoch** | **10s**  **epoch** | **Test for Difference**  **Between Correlations**  *p* value | **60s epoch**  β | **10s epoch**  β | **Test for Equality**  **of Slope**  *p* value |
| --- | --- | --- | --- | --- | --- | --- |
|  | ρ | ρ |
| **Anthropometric Measures** |  |  |  |  |  |  |
| Body weight, lbs | --- | --- | --- | --- | --- | --- |
| Body Mass Index, kg/m^2^ | --- | --- | --- | --- | --- | --- |
| Waist Circumference, cm | --- | --- | --- | --- | --- | --- |
| Whole body fat mass, kg | --- | --- | --- | --- | --- | --- |
| Trunk fat mass, kg | --- | --- | --- | --- | --- | --- |
| Whole body lean mass, kg | --- | --- | --- | --- | --- | --- |
| Trunk lean mass, kg | --- | --- | --- | --- | --- | --- |
| **Bone Parameters** |  |  |  |  |  |  |
| Spine, g/cm^2^ | -0.223^*^ | -0.215^*^ | 0.76 | -30.82^*^ | -32.61^*^ | 0.68 |
| Trochanter, g/cm^2^ | -0.007 | 0.010 | 0.53 | -0.28 | 4.15 | 0.43 |
| Intertrochanter, g/cm^2^ | -0.031 | -0.019 | 0.67 | -3.57 | -1.00 | 0.57 |
| Femoral Neck, g/cm^2^ | -0.021 | 0.022 | 0.11 | -3.70 | 6.19 | 0.09 |
| Hip, g/cm^2^ | -0.026 | -0.009 | 0.52 | -3.47 | 0.72 | 0.43 |
| **Physical Activity** |  |  |  |  |  |  |
| Leisure Physical Activity, MET∙hr∙wk^-1 b^ | 0.321^**^ | 0.321^**^ | 0.99 | 0.41^**^ | 0.47^**^ | 0.18 |
| 400 m walk, s ^d^ | -0.371^***^ | -0.366^***^ | 0.84 | -0.20^***^ | -0.21^***^ | 0.45 |
| **Cardiovascular Disease Risk Factors** |  |  |  |  |  |  |
| Systolic Blood Pressure, mmHg | -0.102 | -0.153 | 0.053 | -0.12 | -0.19 | 0.06 |
| Diastolic Blood Pressure, mmHg | -0.043 | -0.070 | 0.33 | -0.09 | -0.15 | 0.38 |
| Total Cholesterol, mg/dL ^a^ | 0.070 | 0.077 | 0.79 | 0.04 | 0.05 | 0.53 |
| LDL-c, mg/dL ^a^ | 0.054 | 0.069 | 0.58 | 0.04 | 0.05 | 0.38 |
| HDL-c, mg/dL ^a^ | 0.004 | 0.037 | 0.22 | -0.01 | 0.02 | 0.43 |
| Triglycerides, mg/dL ^a^ | 0.058 | -0.011 | 0.01 | 0.03 | 0.01 | 0.07 |
| Insulin, mg/dL ^c^ | -0.228^*^ | -0.242^*^ | 0.59 | -0.96^*^ | -1.02^†^ | 0.66 |
| Glucose, mg/dL ^a^ | -0.136 | -0.153 | 0.52 | -0.24 | -0.29 | 0.41 |

^†^*P*<0.10; ^*^*P*<0.05; ^**^*P*<0.01; ^***^*P*<0.001; ^a^ n=101; ^b^ n=100; ^c^ n=99; ^d^ n=90
